# Long-Term Osteochondral Repair Induced by Electrospun PLA/PCL Scaffolds Functionalized with Polypyrrole and Aggrecan: Histological and Mechanical Evaluation in a Rabbit Model

**DOI:** 10.3390/polym18151906

**Published:** 2026-08-03

**Authors:** Nancy C. Islas-Arteaga, Atlántida M. Raya-Rivera, Juan Morales-Corona, Diego R. Esquiliano-Rendon, Patricia G. Ontiveros-Nevares, Omar E. Uribe-Juárez, Roberto Olayo, María G. Flores Sánchez

**Affiliations:** 1 Department of Electrical Engineering, Universidad Autónoma Metropolitana, Unidad Iztapalapa, Av. San Rafael Atlixco 186, Vicentina, Alc. Iztapalapa, AP 55-534, Mexico City 09310, Mexico; 2 Department of Tissue Engineering, Child Hospital of México Federico Gómez, Dr. Márquez 162, Doctores, Alc. Cuauhtémoc, Mexico City 06720, Mexico; 3 Department of Physics, Universidad Autónoma Metropolitana, Unidad Iztapalapa, Av. San Rafael Atlixco 186, Vicentina, Alc. Iztapalapa, AP 55-534, Mexico City 09310, Mexicooagr@xanum.uam.mx (R.O.); 4 Department of Pathology, Morelense Child Hospital, Av de la Salud No. 1, Emiliano Zapata 62765, Mexico; 5 Vice-Rectory for Research, La Salle University México, Benjamín Franklin 45, Condesa, Alc. Cuauhtémoc, Mexico City 06140, Mexico

**Keywords:** electrospinning, PLA/PCL scaffold, aggrecan, plasma polymerization, osteochondral repair, viscoelastic modeling

## Abstract

Articular cartilage possesses a limited intrinsic repair capacity, and current treatment strategies frequently result in fibrocartilaginous repair tissue with inferior structural and mechanical properties compared with native hyaline cartilage. Increasing evidence indicates that successful restoration of joint function requires regeneration of the entire osteochondral unit and adequate integration between cartilage and subchondral bone. Cartilage tissue engineering has emerged as an effective approach for repairing damaged cartilage. The present study evaluated the long-term performance of electrospun PLA/PCL (70/30) scaffolds coated with iodine-doped polypyrrole (PPy-I), with and without aggrecan incorporation, in a rabbit osteochondral defect model after 12 months of implantation. Two scaffold formulations were evaluated: M1 (PLA-PCL-PPy-I) and M2 (PLA-PCL-PPy-I-AG), each implanted either without cells or after in vitro pre-culture with autologous chondrocytes prior to implantation. Histological analyses were performed to assess tissue organization and osteochondral integration, while indentation testing was used to characterize the mechanical behavior of the regenerated tissues. Experimental force–displacement data were further analyzed using a generalized nonlinear Maxwell viscoelastic model. Histological evaluation revealed that scaffold composition and cellularization influenced the characteristics of the regenerated tissue. The M2 scaffold pre-cultured with autologous chondrocytes exhibited the structural organization most closely resembling native hyaline cartilage, including a tri-zonal architecture and a continuous tidemark indicative of improved osteochondral integration. Mechanical testing demonstrated nonlinear viscoelastic behavior and hysteresis in both regenerated and native tissues. The proposed generalized nonlinear Maxwell viscoelastic model provides a practical framework for the mechanical characterization of regenerated osteochondral tissues using only two effective parameters representing the elastic and viscous response, and may support future studies aimed at estimating their intrinsic mechanical properties.

## 1. Introduction

Articular cartilage is a specialized tissue whose composition and architecture enable it to withstand repetitive compressive loads while facilitating joint motion with a low coefficient of friction. These properties arise from the interaction among the collagen network, proteoglycans (primarily aggrecan), and interstitial water content, which together generate a highly efficient viscoelastic mechanical behavior [1,2]. However, due to its avascular, aneural, and alymphatic nature, its intrinsic repair capacity is limited, particularly in the presence of large defects or deep osteochondral lesions, thereby promoting progression toward structural degeneration and functional impairment [3,4].

In the context of osteoarthritis and other degenerative joint diseases, one of the earliest events is the degradation of aggrecan mediated by members of the A Disintegrin and Metalloproteinase with Thrombospondin Motifs (ADAMTS) family of aggrecanases [5,6]. Aggrecan loss disrupts the organization of the extracellular matrix (ECM), reduces the osmotic pressure required to resist compressive loading, and renders the collagen network more susceptible to proteolytic degradation [5,7,8]. Because aggrecan forms aggregates with hyaluronan and link proteins that stabilize the extracellular matrix, its depletion compromises not only the mechanical integrity of the tissue but also its homeostasis [2,7].

Current clinical strategies for cartilage repair may provide short-term symptomatic improvement; however, they frequently result in the formation of fibrocartilage, a tissue characterized by lower proteoglycan content and inferior mechanical properties compared with native hyaline cartilage [9,10].

Nevertheless, growing evidence indicates that restoration of the articular surface alone may be insufficient to achieve long-term functional repair. Articular cartilage and its supporting bone are tightly coupled and should be viewed as a connected osteochondral unit. Without support from an intact subchondral bed, any treatment directed at the surface chondral lesion is likely to fail [11].

Therapeutic strategies, including microfracture, autologous chondrocyte implantation (ACI), and particulate cartilage implantation techniques, have focused primarily on the structural repair of articular cartilage alone. However, it seems prudent to consider cartilage and subchondral bone as a single entity [12]. From this perspective, the therapeutic goal for large chondral or osteochondral defects should be to restore the physiological properties of the entire osteochondral unit [11]. Consequently, surgeons have sought to restore the osseous phase and the cartilaginous surface together as a functional osteochondral unit [3].

In this context, maintaining the interface at the chondro-osseous junction is a key factor for normal bone growth as well as for the function and maintenance of articular cartilage [13]. The interface between cartilage and bone is a unique region that provides tissue integrity across two structurally distinct materials [14]. As a result, the interplay among the tissues of the osteochondral unit is essential for maintaining joint functionality [13].

Because the function of the osteochondral unit depends on the coordinated interaction among its constituent tissues, characterization of its mechanical response becomes particularly relevant [13]. A better understanding of the mechanical behavior of articular cartilage and the osteochondral unit, together with the quantification of the associated mechanical parameters, is crucial for the development of efficient repair strategies and tissue engineering approaches [13,15].

Tissue engineering is a multidisciplinary field that relies on the interplay among biomaterials, cellular sources, and bioactive signaling molecules to promote functional tissue regeneration [16].

According to Wasyleczko et al., scaffolds for cartilage tissue engineering should provide an appropriate environment for cell adhesion, migration, and development while exhibiting structural and biological features such as porosity, pore interconnectivity, permeability, controlled degradability, adequate mechanical properties, and biocompatibility. In addition, a three-dimensional scaffold architecture is required to prevent chondrocyte dedifferentiation, and pore sizes ranging from 150 to 250 µm are considered suitable for articular chondrocytes [17].

Electrospun scaffolds exhibit high porosity, interconnected pores, and a large surface-area-to-volume ratio, characteristics that have been associated with enhanced cell attachment and proliferation [18]. Poly (lactic acid) (PLA) and polycaprolactone (PCL) have been extensively investigated for orthopedic applications [19,20]. PLA and PCL are biocompatible, biodegradable, and bioabsorbable polymers, and blending PLA with PCL has been reported to provide strong and ductile mechanical properties together with a distinct biodegradation rate [21,22,23].

Nevertheless, electrospun PLA, PCL and PLA/PCL composite membranes are all hydrophobic materials [24]; cells are not able to adhere to the hydrophobic surface of a scaffold, thereby strongly limiting their ability to proliferate and differentiate [25]. The plasma modification technique makes the polymer more hydrophilic, providing the modified surface with high adhesion power and cell proliferation [26]; plasma-polymerized PPy coatings have been shown to improve cell adhesion, survival, reduce dedifferentiation, and enhance cellular differentiation in different cell types [27,28,29].

Chondrocytes are the only cell type present in cartilage and play a crucial role in cartilage formation, growth, repair, and remodeling. Autologous chondrocyte implantation can provide an alternative cell resource with a higher regenerative capacity for cartilage regeneration [30].

Finally, aggrecan, the major proteoglycan in articular cartilage, plays a key structural role in the extracellular matrix. At high concentrations, proteoglycans create a large osmotic swelling pressure that draws water into the tissue, generating a water-swollen matrix that is critical to the biomechanical properties of cartilage. Besides its structural role, aggrecan plays an important role in mediating chondrocyte–chondrocyte and chondrocyte–matrix interactions. Furthermore, aggrecan has been used as a bioactive signal in cartilage tissue engineering scaffolds, while chondroitin sulfate, the major structural component of aggrecan, has been shown to improve chondrogenesis in cartilage tissue engineering applications [2,31].

In a previous pilot study [32], electrospun PLA (70%)/PCL (30%) scaffolds were surface-coated with polypyrrole–iodine (PPy-I) by plasma polymerization. Subsequently, a fraction of the scaffolds was further functionalized by aggrecan (AG) adsorption, generating two experimental groups: PLA/PCL/PPy-I scaffolds (M1) and PLA/PCL/PPy-I/AG scaffolds (M2).

The cytotoxicity of the scaffolds was evaluated by an MTT assay using autologous rabbit chondrocytes cultured on both materials for 7 days. No evidence of significant cytotoxicity was observed during the culture period, as both scaffolds maintained detectable cellular metabolic activity. In addition, aggrecan-functionalized scaffolds (M2) exhibited higher MTT absorbance values than scaffolds without aggrecan (M1) after 7 days of culture.

Additionally, scaffolds seeded with autologous chondrocytes and cultured for 7 days were implanted subcutaneously in the dorsal region of the same rabbit. After 30 days of implantation, the newly formed tissues were retrieved for further characterization.

Histological and immunohistochemical analyses revealed the expression of type II collagen and aggrecan in both neotissues. However, the tissue generated on the aggrecan-functionalized scaffold exhibited a more homogeneous morphology and no apparent evidence of fibrosis, whereas the tissue formed on the scaffold without aggrecan showed regions of fibrous tissue and cells compatible with fibroblasts. The presence of type II collagen and aggrecan in both groups, together with the observation of cells displaying a morphology compatible with chondrocytes, suggests the survival of cells with a chondrogenic phenotype after 30 days of implantation.

Although the previous pilot study demonstrated early chondrogenic activity after one month of subcutaneous implantation, the simultaneous presence of fibrous tissue left unanswered whether this initial response would ultimately mature into hyaline cartilage or progress toward fibrocartilage during long-term implantation. This question motivated the present long-term osteochondral study.

To determine the long-term outcome of this early regenerative response, the present study evaluated the long-term performance of electrospun PLA (70%)/PCL (30%) scaffolds surface-coated with polypyrrole–iodine (PPy-I) by plasma polymerization in a rabbit osteochondral defect model after 12 months of implantation. Two scaffold formulations were compared: M1 (PLA-PCL-PPy-I) and M2 (PLA-PCL-PPy-I-AG), both under conditions with and without autologous chondrocyte seeding.

The study focused on assessing the structural organization of the repair tissue, its integration with the surrounding osteochondral environment, and its functional mechanical behavior. Histological analyses were performed to evaluate tissue architecture and osteochondral integration, whereas indentation testing was used to characterize the mechanical response of the regenerated tissues.

Given the exploratory nature of this study, the work was designed to identify long-term structural and functional trends associated with scaffold composition and cell seeding rather than to establish definitive statistical differences among treatment groups.

Because articular cartilage exhibits a nonlinear viscoelastic mechanical behavior, the ability of a generalized nonlinear Maxwell model to phenomenologically describe the viscoelastic mechanical response of the regenerated tissues was also explored.

## 2. Experiment

### 2.1. Materials

Chloroform, ACS reagent, Ethanol, 200 proof, ACS reagent, ≥99.5%, Pyrrole, reagent grade, 98%, Iodine, ACS reagent, ≥99.8%, and lyophilized aggrecan from bovine articular cartilage (Cat. A1960) were purchased from Sigma-Aldrich (St. Louis, MO, USA).

Ingeo™ Biopolymer 3251D poly(lactic acid) (PLA) was supplied by NatureWorks LLC (Plymouth, MN, USA). According to the manufacturer, this grade exhibits a glass transition temperature ($T_{g}$) of 55–65 °C, a melting temperature ($T_{m}$) of 155–170 °C, and a relative viscosity (RV) of 2.5 (corresponding to an estimated weight-average molecular weight, $M_{w}$, of 70,000–90,000 g/mol). Poly(ϵ-caprolactone) (PCL) with an average molecular weight ($M_{w}$) of ~65,000 g/mol (Cat. No. 181609) was purchased from Sigma-Aldrich (St. Louis, MO, USA). The PCL possesses a nominal melting temperature ($T_{m}$) of 60 °C and a glass transition temperature ($T_{g}$) of 60 °C.

For cell isolation and culture, fetal bovine serum (FBS) and Dulbecco’s Modified Eagle Medium/Nutrient Mixture F-12 (DMEM/F12) were obtained from Gibco, Thermo Fisher Scientific (Waltham, MA, USA). Type II collagenase, L-ascorbic acid (≥99%, crystalline), and antibiotic-antimycotic solution were obtained from Sigma-Aldrich (St. Louis, MO, USA).

Reagents used for animal procedures included Acepromazine maleate (Promazil, Aranda, Queretaro, Mexico), Ketamine hydrochloride (Anesket, PiSA, Guadalajara, Mexico), Sodium pentobarbital (Pisabental, PiSA, Mexico), 2% Lidocaine hydrochloride (Pisacaina, PiSA, Mexico), and Cephalothin sodium (PiSA, Guadalajara, Mexico). Vicryl^®^ absorbable sutures (Ethicon, Johnson & Johnson, Somerville, NJ, USA) were used for surgical wound closure.

For histological processing, paraformaldehyde, ethanol solutions, xylene, paraffin, hematoxylin and eosin were purchased from Sigma-Aldrich, St. Louis, MO, USA. All chemicals were of analytical grade and used as received without further purification.

### 2.2. Ethical Approval for Animal Experiments

The Research, Ethics, and Biosafety Committees of the Federico Gómez Children’s Hospital of Mexico reviewed and approved the protocol HIM/2013/027, entitled “Construction of scaffolds based on polycaprolactone (PCL)–polylactic acid (PLA) coated with polypyrrole–iodine (PPy-I) for cartilage engineering”. All procedures were conducted in accordance with the approved protocol.

The sample size was defined considering the exploratory nature of this long-term preclinical study, the extended 12-month follow-up period, and ethical principles for animal experimentation aimed at minimizing animal use. Accordingly, the selected number of experimental animals was considered sufficient to identify long-term histological and mechanical trends associated with the different scaffold formulations rather than to establish definitive statistical differences among treatment groups. This approach is consistent with recommendations for exploratory preclinical studies and the principle of Reduction within the 3Rs framework [33,34]. Accordingly, the findings of this study should be interpreted as exploratory evidence of the long-term structural and mechanical performance of the evaluated scaffold formulations, providing guidance for future studies with larger experimental groups designed to confirm these observations.

No animals or experimental data were excluded from the study. No additional inclusion or exclusion criteria beyond those defined in the experimental protocol were established a priori.

### 2.3. Scaffold Fabrication

Polymer solutions of PLA and PCL were prepared using a mixture of 90% chloroform and 10% ethanol as a solvent at a concentration of 0.12 g/mL for each polymer. Electrospinning was performed at 30 °C. The PLA and PCL solutions were loaded into two separate syringes and simultaneously injected using an infusion pump at a total flow rate of 2 mL/h, maintaining a 70% (PLA)/30% (PCL) ratio. The solutions were delivered through a needle (positive electrode, 20 kV) with an inner diameter of 0.6 mm toward a rotating stainless-steel collector (15 cm in length and 10 cm in diameter) positioned 20 cm from the needle and rotating at 680 rpm. The resulting fibers formed a nonwoven scaffold composed of nano- and microfibers. Electrospun mats (20 × 10 cm) were then placed under vacuum at 44 °C for 4 days to remove residual solvents.

### 2.4. Plasma Polymerization

The plasma reactor used for pyrrole polymerization consisted of a Pyrex glass tube measuring 20 cm in length, 9 cm in outer diameter, and 0.5 cm in wall thickness, fitted at both ends with stainless-steel caps containing three access ports. A flat circular stainless-steel electrode (6 cm in diameter) was mounted at the central port of each cap and connected to a radiofrequency (RF) power generator to produce the electric field required for plasma discharge. Two ports on one of the caps were used for the introduction of the monomer (pyrrole) and the dopant (iodine), whereas on the opposite cap, one port was connected to the vacuum system and the other to a Pirani gauge for pressure monitoring. Additional details of the experimental setup have been reported previously [32,35].

The electrodes were positioned 8 cm apart, and PLA/PCL scaffolds cut into strips measuring 0.7 × 3 cm were placed between them. An RF power of 20 W at 13.56 MHz was applied, and the reactor pressure was reduced to 1.2 × 10^−1^ Torr. Prior to polymerization, the reactor was purged for 10 min to remove residual impurities.

The vapor-phase monomer was continuously supplied throughout the process, while iodine was introduced for 30 s every 6 min. The total reaction time was 1 h. Upon completion of the treatment, the reactor remained closed for an additional hour in a monomer atmosphere. The system was then vented to atmospheric pressure, and the modified scaffolds (M1, PLA/PCL-PPy-I) were removed from the reactor.

### 2.5. Aggrecan Functionalization of PLA/PCL-PPy-I Scaffolds

An aggrecan (AG) solution was prepared at a concentration of 0.04 mg/mL in distilled water. Four M1 (PLA/PCL-PPy-I) scaffolds were individually placed in 35 × 15 mm Petri dishes and prewetted with 2 mL of the AG solution. Additional solution was then added until the scaffolds were completely submerged, and the samples were maintained for 24 h at room temperature inside a desiccator containing silica gel (without vacuum).

After incubation, the solution was removed and the scaffolds were washed three times with distilled water for 10 min per wash to remove excess aggrecan. Finally, the functionalized scaffolds, designated as M2 (PLA/PCL-PPy-I-AG), were kept under vacuum in a desiccator at room temperature for 24 h to remove residual water.

### 2.6. Cartilage Biopsy and Cell Isolation

Hyaline cartilage biopsies (Figure 1A) were obtained from the xiphoid process of the sternum of male New Zealand White rabbits (2.0–2.5 kg body weight and approximately 3 months of age). The animals were premedicated with acepromazine (9 mg/kg, intramuscularly) and ketamine (20 mg/kg, intramuscularly), and anesthesia was induced by intravenous administration of sodium pentobarbital (20 mg/kg).

Under aseptic conditions, an approximately 1 cm^2^ biopsy was collected from the xiphoid process and transported to the laboratory in sterile Falcon tubes containing PBS supplemented with antibiotic-antimycotic solution (1% *v/v*) (Figure 1B). The xiphoid process was selected as the donor site because it provides an accessible source of autologous chondrocytes while preserving the integrity of the experimental knee joint prior to osteochondral defect creation. The biopsy size was sufficient to obtain the number of cells required for in vitro expansion and subsequent scaffold seeding while minimizing tissue removal from the donor site. In addition, harvesting chondrocytes from an extra-articular site avoids donor-site morbidity associated with articular cartilage biopsy and represents a practical alternative that has been investigated for cartilage tissue engineering and may facilitate future clinical translation of cell-based therapies [36,37,38].

For cell isolation, the tissue was mechanically fragmented and placed in 100 mm culture dishes containing DMEM/F12 supplemented with 10% fetal bovine serum (FBS).

Subsequently, the tissue fragments were subjected to enzymatic digestion with type II collagenase under constant agitation for 4 h (Figure 1C). The resulting supernatants were collected, resuspended in 5 mL of complete medium, and centrifuged to obtain the cell pellet.

The isolated chondrocytes were morphologically evaluated by light microscopy during primary culture. Only passage 1 (P1) chondrocytes were used for scaffold seeding.

### 2.7. Chondrocyte Seeding and Scaffold Preconditioning

PLA/PCL-PPy-I (M1) and PLA/PCL-PPy-I-AG (M2) scaffolds were sterilized by UV irradiation and prehydrated in culture medium for 2 h. Subsequently, the scaffolds were seeded with a chondrocyte suspension at a concentration of 1.2 × 10^6^ cells/mL by adding 2 mL of cell suspension to each scaffold. The samples were incubated for 3 h at 37 °C in a humidified atmosphere containing 5% CO_2_ to promote cell adhesion.

Following cell attachment, 2 mL of DMEM/F12 medium supplemented with 10% fetal bovine serum (FBS), 1% antibiotic-antimycotic solution, and ascorbic acid (0.05 mg/mL) was added to each scaffold. The constructs were maintained in culture for 7 days prior to implantation.

### 2.8. Surgical Implantation Procedure

For the in vivo study, the scaffolds were implanted under aseptic conditions in the knee joint on the articular surfaces of the femur (femoral condyles) and tibia (tibial plateau) of the rabbits’ hind limbs. The animals were premedicated with acepromazine and ketamine administered intramuscularly. General anesthesia was induced by intravenous administration of sodium pentobarbital, and local anesthesia was provided with lidocaine.

The surgical area was shaved (Figure 2A), and a parapatellar incision was performed to expose the joint (Figure 2B,C). At the implantation sites, the articular cartilage was removed using a scalpel (Figure 2D), and scaffolds measuring 0.7 × 0.7 cm were placed in the resulting defects (Figure 2E).

Two rabbits were assigned to the experimental groups, in addition to one healthy control rabbit. In each experimental animal, an M1 scaffold was implanted in one hind limb and an M2 scaffold in the contralateral limb. According to the experimental design (Figure 2F), rabbit 1 received M1 and M2 scaffolds without cells, whereas rabbit 2 received M1 and M2 scaffolds previously seeded with autologous chondrocytes.

After implantation, the surgical wound was closed in two layers using absorbable sutures (Vicryl) (Figure 2G). Finally, the surgical area was disinfected, and a single postoperative dose of cephalothin (11 mg/kg) was administered. The animals were housed individually without immobilization throughout the follow-up period.

### 2.9. Retrieval and Processing of Osteochondral Specimens

After 12 months of implantation, osteochondral specimens corresponding to the PLA/PCL-PPy-I (M1) and PLA/PCL-PPy-I-AG (M2) scaffolds were retrieved. The animals were sedated with acepromazine (5 mg/kg, intramuscularly) and ketamine (20 mg/kg, intramuscularly), and subsequently euthanized by intravenous administration of sodium pentobarbital (40 mg/kg).

A longitudinal incision was made to expose the knee joint (Figure 3A). Subsequently, the osteochondral specimens were harvested by performing cuts through the femur using a manual hacksaw (Figure 3B). Excess soft tissue was removed using scissors and a scalpel, and the intracapsular ligaments, as well as the medial and lateral collateral ligaments, were sectioned to separate the femoral and tibial segments.

The retrieved specimens were placed in Falcon tubes containing F12 medium and transported at 4 °C for subsequent processing. The femoral segments, containing the femoral condyles and regenerated tissue, were used for compression mechanical testing. The tibial segments, including the tibial plateau and regenerated tissue, were fixed and processed for histological evaluation.

### 2.10. Histological Processing of Osteochondral Specimens

Osteochondral specimens retrieved from M1 and M2 scaffolds, with and without chondrocytes, were fixed in paraformaldehyde and processed using conventional histological techniques for paraffin embedding.

The samples were dehydrated through a graded ethanol series (70, 80, 96, and 100%) under constant agitation, with solution changes every 12 h to ensure complete removal of tissue water. Subsequently, the specimens were cleared in xylene and infiltrated with paraffin.

The resulting paraffin blocks were sectioned at a thickness of 5 μm, and the histological sections were stained with hematoxylin and eosin (H&E) for general morphological evaluation of the tissues.

### 2.11. Indentation Compression Testing

Indentation compression tests were performed at room temperature using a CT3-4500 Texture Analyzer (AMETEK Brookfield, Middleboro, MA, USA) equipped with a TA39 cylindrical flat-ended indenter (Ø 2 mm). Osteochondral specimens were maintained hydrated in F12 medium at 4 °C until testing.

For mechanical testing, the femoral segments were secured to the base of the instrument using a holding clamp, aligning the articular surface beneath the indenter (Figure 4A). To improve stability during testing, the femurs were sectioned longitudinally using a manual hacksaw (Figure 4B).

All tests were conducted at a displacement rate of 0.5 mm/min with a preload of 0.1 N and a single loading–unloading cycle. For the retrieved M1 and M2 specimens, indentations were performed on both the medial and lateral regions of the regenerated articular surface while avoiding the tissue edges. The target indentation depth was 0.7 mm for all retrieved specimens.

Additionally, reference indentation tests were performed on native articular cartilage obtained from a healthy rabbit. In this case, measurements were acquired from the lateral femoral condyle using indentation depths of 0.3 mm and 0.5 mm.

Because the original articular cartilage had been removed during the surgical procedure to expose the subchondral bone, and considering the risk of delamination associated with manipulation of the regenerated tissue, direct measurements of tissue thickness were not performed. Therefore, the mechanical tests were interpreted as an evaluation of the overall mechanical response of the retrieved osteochondral unit.

The indentation depths selected for native cartilage (0.3 and 0.5 mm) were chosen considering the reported thickness of rabbit femoral articular cartilage. In contrast, the 0.7 mm indentation depth used for regenerated specimens was selected to evaluate the mechanical response of the repaired osteochondral unit rather than the cartilage layer alone [39].

Force–displacement curves corresponding to the loading and unloading phases were recorded during each test for subsequent analysis and mechanical modeling.

### 2.12. Nonlinear Maxwell Viscoelastic Model

The Maxwell model is one of the most widely used viscoelastic models for describing the mechanical behavior of articular cartilage [15]. Since articular cartilage exhibits nonlinear viscoelastic mechanical behavior [15], a nonlinear generalization of the Maxwell model (Figure 5) was employed in this work to describe the mechanical response of the samples based on the experimental data obtained from the indentation compression tests.

The constitutive equations of each element are given by

$$F_{k}=E_{k}{\left(x_{ks}\right)}^{k}$$

$$F_{\kappa }=\eta_{k}{\left({\dot{x}}_{kd}\right)}^{k}$$

where $F_{k}$ represents the force associated with the k-th Maxwell element; $E_{k}$ and $\eta_{k}$ correspond to the elastic and viscous constants of order k, with units of $Nm^{-k}$ and $Nm^{-k}s^{k}$, respectively; while $x_{ks}$ and $x_{kd}$ represent the displacements associated with the spring and dashpot of the k-th element.

In this formulation, each Maxwell element incorporates a viscoelastic contribution of order (k).

Since multicollinearity can compromise the stability and accuracy of parameter estimation [40], and considering that many constitutive laws proposed to describe the stress–strain behavior of soft tissues are characterized by the presence of an exponential function [41]. Under these considerations, the constants $E_{k}$ and $\eta_{k}$ can be expressed as functions of the constants $E_{1}$ and $\eta_{1}$, and therefore, the model converges to a formulation described by only two parameters:

$$F_{T}\left(t\right)=F_{1}\left(t\right)e^{{C_{1}F}_{1}\left(t\right)}$$

with

$$F_{1}\left(t\right)=\eta_{1}*\dot{x}\;+C_{0}e^{-\frac{t}{\tau_{1}}}$$

where $C_{0}$ is determined by the initial conditions, $C_{1}$ is a unit-magnitude constant with units of $N^{-1}$, and $\tau_{1}=\eta_{1}/E_{1}$.

### 2.13. Parameter Estimation and Data Processing

Experimental data were fitted to the generalized nonlinear Maxwell model using Python 3.10.19 and the Pandas 2.3.3, NumPy 1.26.4, and SciPy 1.12.0 libraries.

Model parameters were estimated using the curve fit function from the SciPy library, which determines the optimal parameters by minimizing the sum of squared residuals between the experimental data and the response predicted by the model. As output, the function returns the optimal parameter vector and the covariance matrix associated with the fitted parameters.

The quality of each fit was evaluated using the coefficient of determination ($R^{2}$) calculated as

$$R^{2}=1-\frac{\sum_{i=1}^{n}{\left(y_{i}-{\hat{y}}_{i}\right)}^{2}}{\sum_{i=1}^{n}{\left(y_{i}-\bar{y}\right)}^{2}}$$

where $y_{i}$ represents the experimental data, ${\hat{y}}_{i}$ the values predicted by the model, and $\bar{y}$ the mean value of the experimental data.

Parameter uncertainties were estimated from the covariance matrix obtained during the fitting procedure by calculating the square root of its diagonal elements.

For the unloading phase, only data points with force values greater than the instrumental uncertainty of the texture analyzer (0.22 N) were considered during the fitting procedure. The first data point below this threshold was retained as a reference point, whereas subsequent points were excluded.

Physical constraints were incorporated into the fitting procedure for the unloading phase through the viscous parameter of the model. Since the volume change in articular cartilage during compression is caused by fluid loss, and part of its viscous response is associated with fluid flow through its porous structure [15], a constraint was imposed such that the viscosity estimated during unloading could not exceed the viscosity determined during loading plus its associated uncertainty.

## 3. Results and Discussion

### 3.1. Histologies

Figure 6 shows a representative micrograph of a transverse histological section of articular cartilage from the control group, stained with hematoxylin and eosin (H&E). The overall structural organization is consistent with that reported in the literature for healthy articular cartilage [15,42].

In the superficial cartilage zone (SCZ), flattened chondrocytes are arranged parallel to the articular surface, appearing as pairs of cells, and individual cells dispersed within these patterns. In contrast, the middle and deep zones contain double or multiple chondrons organized as vertical columns of cells [43].

The staining pattern of the extracellular matrix (ECM) suggests an arcade-like architecture compatible with Benninghoff’s model, with obliquely and randomly oriented fibrillar structures [15,44].

A prominent basophilic line corresponding to the tidemark is clearly identified. The tidemark distinguishes the deep zone from the mature calcified cartilage and appears histologically as a well-defined basophilic interface [45,46]. The irregular geometry of the tidemark is associated with resistance to shear stresses [45].

Calcified cartilage (CC) plays an integral role in anchoring the articular cartilage to the underlying subchondral bone (SB) by securing the collagen fibrils of the deep zone, thereby promoting optimal structural integration stresses [45]. In this context, the CC acts as a force transmitter and contributes to mechanical stabilization [42,45,46].

Finally, a region corresponding to the synovial capsule (SC) is also observed. These findings provide the histological reference for comparison with the regenerated tissues presented below.

Figure 7A shows the neocartilage formed following implantation of the M1 scaffold in the osteochondral defect model. The regenerated tissue exhibited features consistent with fibrocartilage, including a discontinuous tidemark, suggesting incomplete structural integration between the newly formed tissue and the subchondral bone.

This outcome was partially expected. However, the M1 scaffold incorporated several characteristics considered desirable for articular cartilage tissue engineering [17], including a three-dimensional electrospun fibrous architecture, interfiber spaces of 156.17 ± 85.57 μm^2^, interconnected pores, and surface functionalization for cell adhesion. Morphological characterization further revealed a bimodal fiber diameter distribution (1.43 ± 0.35 μm and 3.16 ± 0.65 μm), while FTIR analysis confirmed the presence of the PLA/PCL fibrous framework and surface functionalization with plasma-polymerized PPy [32]. This type of coating has been reported to promote cell adhesion, enhance cell survival, reduce dedifferentiation processes, and support cellular differentiation in different cell types [27,28,29].

As discussed previously, tissue engineering relies on the interplay among biomaterials, cellular sources, and bioactive signaling molecules to achieve functional tissue regeneration [16]. Consequently, when only one of these three components was employed, the use of the M1 scaffold as a standalone regenerative strategy was insufficient to promote the regeneration of tissue exhibiting histological characteristics comparable to those of native articular cartilage.

The formation of fibrocartilage observed in the M1 group indicates that, although the defect was filled with newly formed tissue, regeneration of articular cartilage with characteristics equivalent to those of native tissue was not achieved. Traditional cartilage repair procedures frequently result in the formation of fibrocartilage, a tissue with inferior mechanical properties and biological functions compared with those of hyaline cartilage. Furthermore, fibrocartilage formed within cartilage defects lacks the functional characteristics of native articular cartilage and has been associated with the progression of degenerative joint processes. The presence of fibrocartilage may be related to the normal tissue repair processes that occur following an osteochondral injury, in which cellular activation associated with wound healing promotes collagen deposition and the formation of fibrous tissue [47].

Figure 7B, corresponding to the M1 scaffold pre-seeded with chondrocytes, shows a tissue with a heterogeneous morphology and irregular organization. This organization differs from that observed in the neotissue formed when the M1 scaffold was implanted without chondrocytes. The incorporation of chondrocytes may have contributed to tissue remodeling processes within the defect. However, the observed organization does not correspond to the characteristic architecture of native hyaline cartilage, and regions consistent with fibrocartilage can still be identified.

This result was also partially expected and is consistent with the observations obtained in the pilot study [32], in which chondrocyte-seeded scaffolds exhibited type II collagen and aggrecan expression following implantation. Nevertheless, regions of fibrous tissue and cells with a morphology compatible with fibroblasts were also observed. Previous studies have reported that chondrocytes may undergo dedifferentiation when exposed to inflammatory microenvironments or non-native physical environments, whereas maintenance of the chondrocyte phenotype has been associated with an appropriate three-dimensional and mechanical microenvironment [48,49]. Therefore, although the initial presence of chondrocytes may have promoted processes associated with cartilage regeneration, the results suggest that this strategy was insufficient to promote the formation of tissue with characteristics comparable to those of native articular cartilage.

Figure 7C shows the neocartilage formed following implantation of the M2 scaffold in the osteochondral defect model. Greater homogeneity in neotissue thickness was observed compared with the tissues generated using the M1 scaffold. In addition, a region of fibrocartilage was identified near the articular surface. The absence of a well-defined tidemark suggests that integration with the subchondral bone has not yet been achieved. Overall, this neocartilage has not yet reached a structural state comparable to that of native articular cartilage.

The formation of a tissue exhibiting a lower degree of fibrosis compared with that observed in the M1 group constitutes an unexpected finding, given that no chondrocytes were incorporated into the scaffold prior to implantation. The greater homogeneity observed in neotissue thickness in the M2 group is likely related to the adsorption of aggrecan onto the scaffold surface. As the major proteoglycan of articular cartilage, aggrecan contributes to the generation of osmotic swelling pressure that draws water into the tissue, generating a water-swollen matrix [2,31].

However, although the generated tissue exhibited a more uniform thickness and a lower presence of fibrotic tissue in the middle and deep regions of the defect, regions compatible with fibrocartilage were still identified in the superficial zone, and a well-defined tidemark was not observed. Taken together, these results suggest that the incorporation of aggrecan may contribute to the development of neotissue with more homogeneous thickness and a lower degree of fibrosis; nevertheless, aggrecan alone was insufficient to promote the regeneration of tissue with structural characteristics equivalent to those of native articular cartilage.

The M2 sample pre-cultured with chondrocytes (Figure 7D) displayed the structural organization most similar to native hyaline cartilage. In this group, a tri-zonal arrangement is observed, with cellular presence in all three regions of the neotissue and a distribution morphologically comparable to that seen in healthy cartilage. Furthermore, a continuous and homogeneous tidemark is identified, consistent with a more advanced structural integration with the subchondral bone.

This result was partially expected, as the M2 scaffold pre-seeded with chondrocytes simultaneously incorporated the three fundamental components of a tissue engineering strategy: a scaffold with structural characteristics favorable for articular cartilage regeneration, a cellular source capable of participating in the formation, repair, and remodeling of cartilaginous tissue, and a biomolecule with structural and cell-signaling functions.

Chondrocytes play a fundamental role in the synthesis and remodeling of the cartilage extracellular matrix [30], whereas aggrecan, in addition to contributing to the maintenance of a highly hydrated matrix, participates in chondrocyte–chondrocyte and chondrocyte–matrix interactions and has been employed as a signaling molecule to promote chondrogenic processes [2,31]. The combination of these elements likely contributed to the development of a tissue with a structural organization more closely resembling that of native articular cartilage.

In addition to exhibiting a tissue organization and cellular distribution comparable to those of native articular cartilage, this group displayed a continuous and homogeneous tidemark, suggesting integration between the neocartilage and the subchondral bone. This feature is particularly relevant in the repair of osteochondral defects, where restoration of the cartilage–bone interface constitutes a fundamental requirement for structural tissue recovery.

Overall, the histological findings indicate that none of the regeneration strategies evaluated in this study, except for the aggrecan-functionalized scaffold pre-seeded with autologous chondrocytes, was sufficient to promote the formation of tissue structurally comparable to native hyaline cartilage. Although the electrospun PLA/PCL scaffold provided a porous, interconnected three-dimensional architecture with physicochemical characteristics favorable for articular cartilage regeneration, and aggrecan contributed structural properties together with biological cues characteristic of the native extracellular matrix, these elements alone were insufficient to promote the regeneration of hyaline cartilage. Likewise, the incorporation of autologous chondrocytes in the absence of aggrecan resulted only in partial tissue remodeling, suggesting that an appropriate extracellular microenvironment is required to support maintenance of the chondrogenic phenotype. Therefore, only the combined incorporation of the scaffold, aggrecan, and autologous chondrocytes was likely sufficient to provide the structural, biological, and cellular components required to promote the formation of repair tissue more closely resembling native articular cartilage.

The histological findings should be interpreted considering the exploratory nature of the present study. Because only one rabbit was included in each experimental group, the observed biological responses cannot be generalized or considered statistically representative of treatment effects.

### 3.2. Mechanical Test

The force–displacement curves obtained from the mechanical compression tests for both the neocartilage samples (medial and lateral regions) and the healthy rabbit hyaline cartilage are shown in Figure 8. In general, a nonlinear behavior was observed, characterized by distinct loading and unloading trajectories, giving rise to the formation of closed loops.

The obtained results are consistent with previous reports for articular cartilage, where a nonlinear stress–strain relationship accompanied by hysteresis has been described [50]. The hysteretic behavior of articular cartilage is characterized by the difference between the loading and unloading phases of the stress–strain curve, which describes a loop. This hysteresis is partly the result of time-dependent behavior and may also be associated with microstructural or molecular rearrangements. The area enclosed within these loops represents the energy dissipated and/or stored by the sample during loading [15].

The mechanical testing data were fitted using a generalized Maxwell model with nonlinear responses. The parameters obtained for the loading and unloading phases, together with their corresponding uncertainties, are presented in Table 1. Since the mechanical tests were performed using force–displacement curves, the obtained parameters represent effective mechanical properties of the osteochondral unit and therefore depend on the geometry and dimensions of the sample. In future studies, the incorporation of geometric measurements will allow the transformation of the data into stress–strain curves in order to estimate the intrinsic mechanical properties of articular cartilage and compare the results with values reported in the literature.

The coefficients of determination ($R^{2}$) obtained were, for the most part, greater than or equal to 0.98. During the loading phase, all fits presented $R^{2}$ values greater than or equal to 0.95. During the unloading phase, all $R^{2}$ values obtained were greater than or equal to 0.94, with the exception of a single case that presented an $R^{2}$ value of 0.88.

Nevertheless, $R^{2}$ values close to one do not necessarily guarantee that the estimated parameters are reliable. As can be observed in Table 1, during the loading phase the percentage uncertainties of the parameters were lower than 1.5%, whereas during the unloading phase the percentage uncertainties were considerably higher, with values predominantly ranging between 10% and 40%. These results indicate that, although the model adequately reproduces the overall trend of the experimental data, the parameters were estimated with lower precision during the unloading phase.

Some authors have proposed that fluid exudation from articular cartilage may promote the formation of a vacuum between the sample and the indenter. This phenomenon may generate a suction response similar to adhesive behavior and introduce artifacts during the unloading phase of indentation tests [51]. Consequently, these artifacts could contribute to the increase in the uncertainties observed in the parameters estimated during the unloading phase.

In the data corresponding to the unloading phase (Figure 8), a tail of data points can be observed whose values fall below the instrumental uncertainty of the measurement system (0.22 N). Consequently, a fraction of the mechanical response of the samples cannot be distinguished from instrumental noise, implying a loss of experimental information during the final stage of unloading.

To evaluate this loss of information, the number of data points with values above the instrumental uncertainty during the unloading phase (N) was plotted as a function of the percentage uncertainty associated with η. As shown in Figure 9A, an approximately linear trend can be observed between both variables. To quantify this relationship, the Pearson correlation coefficient was calculated between N and the percentage uncertainties of the parameters E and η (Figure 9B).

The variables that exhibited the highest correlation were N and the percentage uncertainty associated with η. This negative correlation is consistent with the physical interpretation of the parameter η, which represents the time-dependent mechanical response of the tissue [15]. As N increases, a greater number of data points are available to characterize mechanical relaxation during the unloading phase, allowing η to be estimated with greater precision. Conversely, as N decreases, the amount of experimental information available regarding this behavior is reduced, increasing the uncertainty associated with this parameter.

The next most strongly correlated variables were the percentage uncertainties of the parameters E and η. The observed positive correlation indicates that an increase in the uncertainty associated with η tends to be accompanied by an increase in the uncertainty associated with E. This suggests that when the available experimental information is insufficient to adequately characterize the time-dependent response of the tissue, the precision with which its effective stiffness can be estimated also decreases.

Finally, the variables that exhibited the weakest correlation were N and the percentage uncertainty associated with E. This result is also consistent with the physical interpretation of the model, since the elastic response of articular cartilage is commonly classified as a time-independent behavior [15]. Consequently, the loss of information associated with an incompletely captured relaxation process should primarily affect the estimation of the viscous parameters, whereas its effect on the elastic parameters would be comparatively smaller.

To conclude the analysis of the fitting results, the obtained parameters were examined. First, the parameter magnitudes were found to fall within comparable ranges across the different samples, with no clearly outlying values observed. In addition, different parameter values were obtained for the loading and unloading phases. This behavior is consistent with the hysteretic nature of articular cartilage, in which the mechanical response during unloading differs from that observed during loading. Hysteresis has been proposed to be associated with time-dependent mechanisms, as well as with possible microstructural or molecular rearrangements within the extracellular matrix [15]. Nevertheless, since the obtained parameters correspond to effective mechanical properties derived from force–displacement curves, a more detailed interpretation of these results and of the differences observed between loading and unloading is left for future studies in which intrinsic mechanical properties derived from stress–strain curves are available.

A limitation of the mechanical characterization is that each mechanical evaluation consisted of a single loading–unloading indentation cycle. Consequently, the variability associated with test repeatability could not be assessed, and the uncertainties reported for the model parameters correspond to those estimated from the nonlinear regression used to fit the proposed viscoelastic model. Accordingly, the interpretation of the mechanical results should consider these methodological limitations.

## 4. Conclusions

The present study evaluated the long-term regenerative performance of electrospun PLA/PCL scaffolds functionalized with plasma-polymerized polypyrrole and aggrecan, with and without autologous chondrocyte seeding, in a rabbit osteochondral defect model after 12 months of implantation. This long-term evaluation was designed to determine the outcome of the early regenerative response observed in our previous pilot study and to assess whether this response evolved toward the regeneration of tissue structurally resembling native hyaline cartilage, together with its osteochondral integration and mechanical behavior through viscoelastic modeling.

After 12 months of implantation, only the aggrecan-functionalized scaffold pre-seeded with autologous chondrocytes promoted the formation of repair tissue with structural characteristics closely resembling native hyaline cartilage, including a trilaminar organization and a continuous tidemark suggestive of improved osteochondral integration. In contrast, the remaining regeneration strategies produced tissues exhibiting different degrees of fibrocartilage formation and failed to regenerate tissue structurally comparable to native articular cartilage.

The present findings are consistent with current trends in cartilage tissue engineering, where effective cartilage regeneration is increasingly pursued through multifactorial strategies that integrate cells, biomaterials, and bioactive cues rather than single-component solutions. Furthermore, recent advances indicate a technological transition from structural replacement toward functional reconstruction, emphasizing the importance of restoring tissue architecture and function. Despite these advances, the clinical translation of cartilage tissue engineering remains challenging. Although substantial progress has been achieved in basic research, promising in vivo findings do not always translate into durable clinical benefit, and clinical translation continues to be limited [52,53].

The generalized Maxwell model with nonlinear response provided a phenomenological description of the mechanical response of regenerated and native osteochondral tissues during indentation testing. Although the estimated parameters correspond to effective mechanical properties derived from force–displacement data, the proposed methodology establishes a quantitative framework for comparing the mechanical behavior of different osteochondral tissues under equivalent experimental conditions and may serve as a reference for future developments aimed at estimating intrinsic material properties.

The present findings should be interpreted considering the exploratory nature of the study. Because only one rabbit was included in each experimental group, the biological findings cannot be generalized or considered statistically representative of treatment effects. In addition, the absence of intermediate implantation time points precludes determination of when osteochondral regeneration occurred, while the mechanical characterization was limited to a single loading–unloading indentation cycle for each evaluation, precluding assessment of measurement variability.

Despite these limitations, the proposed tissue engineering strategy and the viscoelastic modeling approach provide a foundation for future studies on long-term osteochondral regeneration and quantitative mechanical characterization.

## Figures and Tables

**Figure 1:**
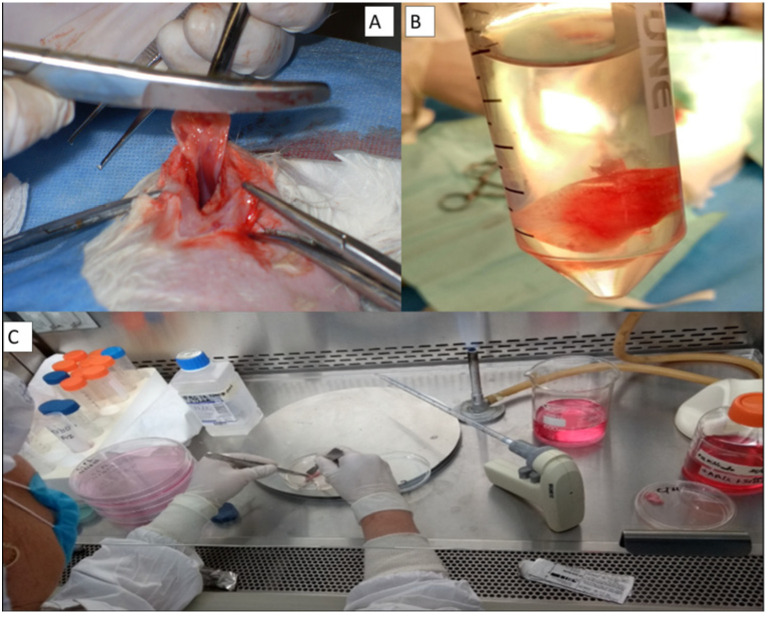
(**A**) Biopsy taken of the xiphoid appendage of the cartilage sternum. (**B**) Washing and transporting the sample. (**C**) Processing the sample: mechanical disintegration for cell multiplication.

**Figure 2:**
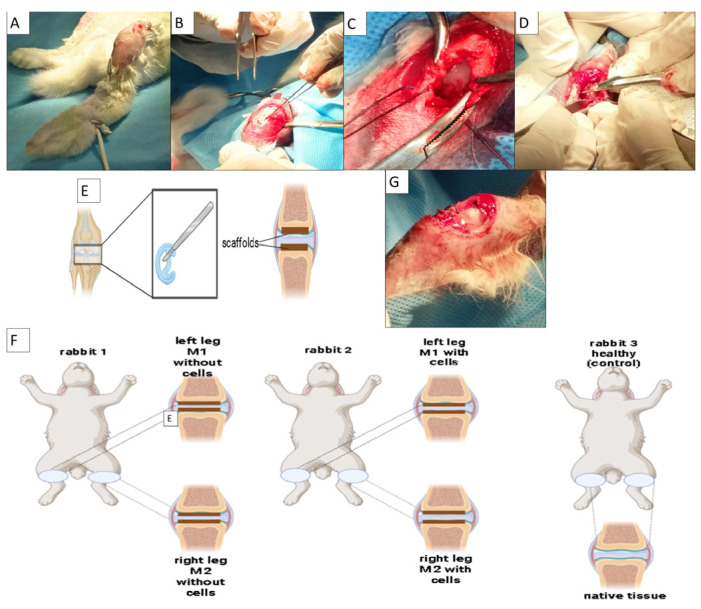
Surgical procedure for scaffold implantation in the rabbit knee joint. (**A**) General anesthesia and preparation of the surgical area. (**B**) Parapatellar incision. (**C**) Exposure of the joint surface. (**D**) Removal of the articular cartilage using a scalpel. (**E**) Placement of the scaffold at the implantation site. (**F**) Experimental design and distribution of the scaffolds among the animals. (**G**) Surgical wound closure.

**Figure 3:**
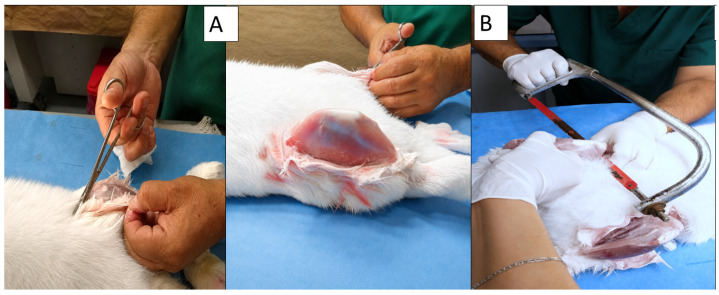
Retrieval of osteochondral specimens after 12 months of implantation. (**A**) Incision and removal of the skin to expose the knee joint. (**B**) Sectioning of the femur to harvest the osteochondral specimen.

**Figure 4:**
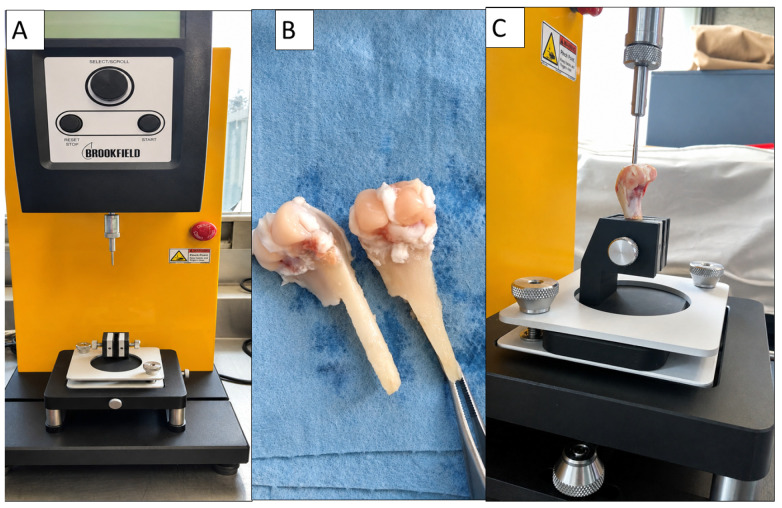
Experimental setup for indentation compression testing. (**A**) Brookfield CT3-4500 texture analyzer used for mechanical testing. (**B**) Longitudinally sectioned femoral specimens prepared for testing. (**C**) Mounting and alignment of the osteochondral specimen beneath the indenter during the compression test.

**Figure 5:**
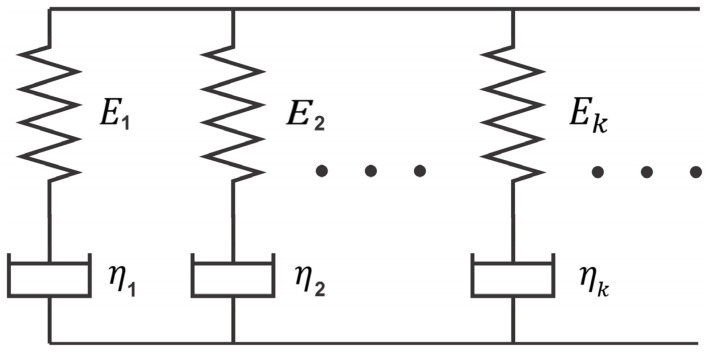
Shows the mechanical representation of the model, consisting of a series of Maxwell elements connected in parallel.

**Figure 6:**
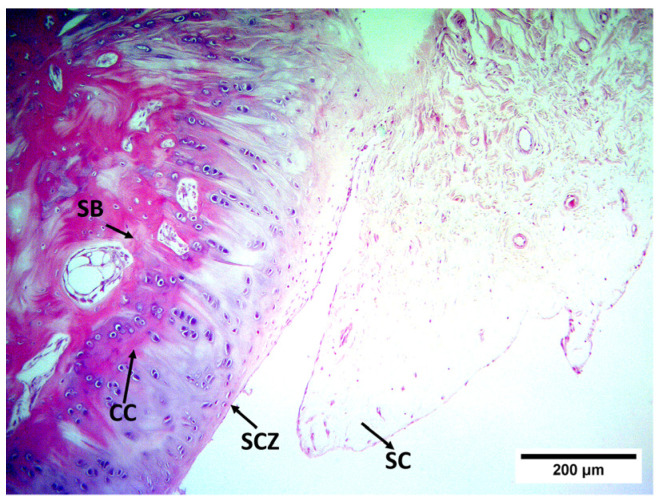
Histology of native rabbit hyaline cartilage (control). SB—subchondral bone; CC—calcified cartilage; SCZ—superficial cartilage zone; SC—synovial capsule.

**Figure 7:**
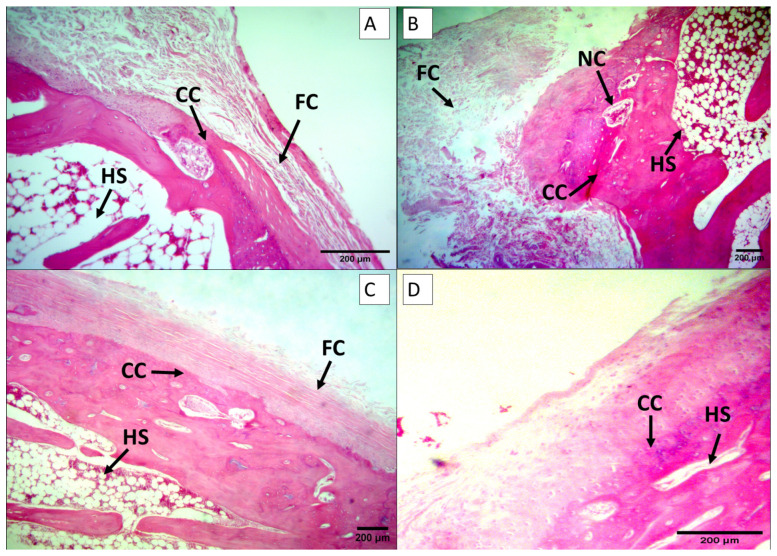
Histology of neotissues generated on (**A**) M1 without cells, (**B**) M1 with cells, (**C**) M2 without cells, and (**D**) M2 with cells. FC—fibrocartilage; CC—calcified cartilage; HS—subchondral bone; NC—calcified nodule.

**Figure 8:**
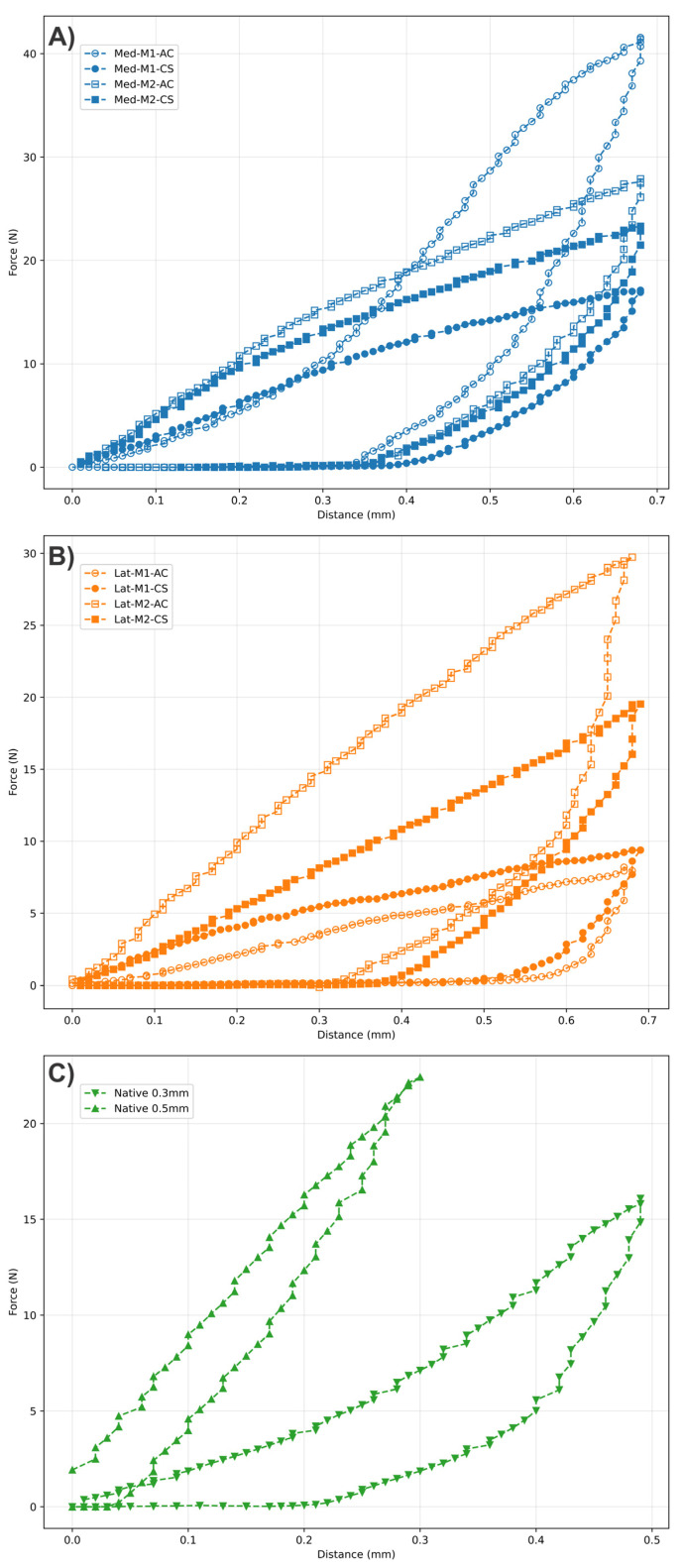
Data obtained from the mechanical compression tests of the neocartilage samples in the medial region (**A**), lateral region (**B**), and healthy rabbit hyaline cartilage in the lateral region (**C**). The nomenclature used was: Med = medial, Lat = lateral, AC = acellular, and CS = cell-seeded.

**Figure 9:**
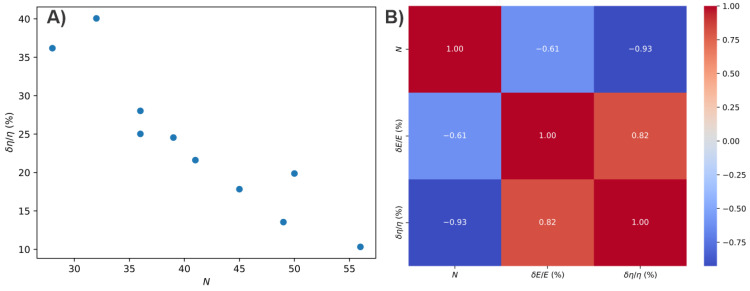
(**A**) Relationship between the number of data points with values above the instrumental uncertainty during the unloading phase (N) and the percentage uncertainty of the parameter η. (**B**) Heat map of the Pearson correlation coefficient between N and the percentage uncertainties of the parameters E and η.

**Table 1:** Parameters and uncertainties obtained by fitting the mechanical testing data to the generalized Maxwell model with nonlinear responses. The subscripts l and u denote the parameters and uncertainties corresponding to the loading and unloading phases, respectively.

Sample	$E_{l}\left(k\frac{N}{m}\right)$	$\frac{\delta E_{l}}{E_{l}}$ (%)	$\eta_{l}\left(k\frac{Ns}{m}\right)$	$\frac{\delta \eta_{l}}{\eta_{l}}$ (%)	$E_{u}\left(k\frac{N}{m}\right)$	$\frac{\delta E_{u}}{E_{u}}$ (%)	$\eta_{u}\left(k\frac{Ns}{m}\right)$	$\frac{\delta \eta_{u}}{\eta_{u}}$ (%)
Med-M1-AC	8.93	0.24	392.25	0.18	3.28	4.22	392.94	10.32
Med-M1-CS	11.73	0.58	254.75	0.17	3.04	10.81	255.18	24.55
Med-M2-AC	14.94	0.34	289.4	0.08	2.62	7.6	183.21	13.54
Med-M2-CS	14.7	0.42	273.43	0.1	2.1	11.17	131.83	17.83
Lat-M1-AC	5.94	1.5	211.88	0.9	2.47	32.76	30.92	40.07
Lat-M1-CS	9.88	1.17	201.81	0.32	4.58	15.52	202.46	36.18
Lat-M2-AC	13.43	0.32	300.63	0.09	1.13	16.53	48.07	19.87
Lat-M2-CS	9.09	0.55	275.05	0.24	2.94	9.36	275.71	21.61
Native 0.5 mm	7.58	0.91	334.69	1.08	2.49	16.63	95.73	25.03
Native 0.3 mm	27.79	0.63	274.84	0.18	2.53	12.77	275.35	28.03

## Data Availability

The original contributions presented in this study are included in the article. Further inquiries can be directed to the corresponding authors.

## References

[1] Roughley P.J. (2006). The Structure and Function of Cartilage Proteoglycans. Eur. Cell. Mater..

[2] Kiani C., Chen L., Wu Y.J., Yee A.J., Yang B.B. (2002). Structure and Function of Aggrecan. Cell Res..

[3] Ai C., Lee Y.H.D., Tan X.H., Tan S.H.S., Hui J.H.P., Goh J.C.H. (2021). Osteochondral Tissue Engineering: Perspectives for Clinical Application and Preclinical Development. J. Orthop. Transl..

[4] Aswathy J., Joseph J., Abraham A. (2024). Challenges in Osteochondral Repair—A Critical Review. Biofunct. Mater..

[5] Pratta M.A., Yao W., Decicco C., Tortorella M.D., Liu R.Q., Copeland R.A., Magolda R., Newton R.C., Trzaskos J.M., Arner E.C. (2003). Aggrecan Protects Cartilage Collagen from Proteolytic Cleavage. J. Biol. Chem..

[6] Nagase H., Kashiwagi M. (2003). Aggrecanases and Cartilage Matrix Degradation. Arthritis Res. Ther..

[7] Roughley P.J., Mort J.S. (2014). The Role of Aggrecan in Normal and Osteoarthritic Cartilage. J. Exp. Orthop..

[8] Karsdal M.A., Madsen S.H., Christiansen C., Henriksen K., Fosang A.J., Sondergaard B.C. (2008). Cartilage Degradation Is Fully Reversible in the Presence of Aggrecanase but Not Matrix Metalloproteinase Activity. Arthritis Res. Ther..

[9] Brain J., Alini M., Cucchiarini M., Dodge G.R., Eglin D., Guilak F., Madry H., Mata A., Mauck R.L., Semino C.E. (2013). Tissue Engineering for Articular Cartilage Repair—The State of the Art. Eur. Cell. Mater..

[10] Moran C.J., Ramesh A., Brama P.A.J., O’Byrne J.M., O’Brien F.J., Levingstone T.J. (2016). The Benefits and Limitations of Animal Models for Translational Research in Cartilage Repair. J. Exp. Orthop..

[11] Gomoll A.H., Madry H., Knutsen G., van Dijk N., Seil R., Brittberg M., Kon E. (2010). The Subchondral Bone in Articular Cartilage Repair: Current Problems in the Surgical Management. Knee Surg. Sports Traumatol. Arthrosc..

[12] Lepage S.I.M., Robson N., Gilmore H., Davis O., Hooper A., St John S., Kamesan V., Gelis P., Carvajal D., Hurtig M. (2019). Beyond Cartilage Repair: The Role of the Osteochondral Unit in Joint Health and Disease. Tissue Eng. Part B Rev..

[13] Berni M., Marchiori G., Baleani M., Giavaresi G., Lopomo N.F. (2024). Biomechanics of the Human Osteochondral Unit: A Systematic Review. Materials.

[14] Kazemi M., Williams J.L. (2020). Properties of Cartilage–Subchondral Bone Junctions: A Narrative Review with Specific Focus on the Growth Plate. Cartilage.

[15] Petitjean N., Canadas P., Royer P., Noël D., Le Floc’h S. (2023). Cartilage Biomechanics: From the Basic Facts to the Challenges of Tissue Engineering. J. Biomed. Mater. Res. A.

[16] Farjaminejad R., Farjaminejad S., Garcia-Godoy F., Marya A., Nucci L., Jamilian A. (2026). Advances and Challenges in Tissue Engineering: Biomaterials, Cellular Strategies, and Clinical Applications. J. Funct. Biomater..

[17] Wasyłeczko M., Sikorska W., Chwojnowski A. (2020). Review of Synthetic and Hybrid Scaffolds in Cartilage Tissue Engineering. Membranes.

[18] Rnjak-Kovacina J., Weiss A.S. (2011). Increasing the Pore Size of Electrospun Scaffolds. Tissue Eng. Part B Rev..

[19] Athanasiou K.A., Darling E.M., Hu J.C., Almarza A.A., Detamore M.S., Kalpakci K.N., Sanchez-Adams J. (2009). Articular Cartilage Tissue Engineering.

[20] Xiang Z. (2017). Science and Principles of Biodegradable and Bioresorbable Medical Polymers.

[21] Meng C. (2025). Electrospinning PLLA/PCL Blend Fibre-Based Materials and Their Biomedical Application: A Mini Review. Polymers.

[22] Kanmaz D., Aylin Karahan Toprakci H., Olmez H., Toprakci O. (2018). Electrospun Polylactic Acid Based Nanofibers for Biomedical Applications. Mater. Sci. Res. India.

[23] Łysik D., Deptuła P., Chmielewska S., Bucki R., Mystkowska J. (2022). Degradation of Polylactide and Polycaprolactone as a Result of Biofilm Formation Assessed under Experimental Conditions Simulating the Oral Cavity Environment. Materials.

[24] Lu Y., Chen Y.C., Zhang P.H. (2016). Preparation and Characterisation of Polylactic Acid (PLA)/Polycaprolactone (PCL) Composite Microfibre Membranes. Fibres Text. East. Eur..

[25] Teimouri R., Abnous K., Taghdisi S.M., Ramezani M., Alibolandi M. (2023). Surface Modifications of Scaffolds for Bone Regeneration. J. Mater. Res. Technol..

[26] Paterlini T.T., Nogueira L.F.B., Tovani C.B., Cruz M.A.E., Derradi R., Ramos A.P. (2017). The Role Played by Modified Bioinspired Surfaces in Interfacial Properties of Biomaterials. Biophys. Rev..

[27] Alvarado Muñoz E.J., Olayo González R., Olayo González M.G., Cruz Cruz G.J., Uribe Juárez O.E., Coyoy Salgado A., Martinez Fong D., Salgado Ceballos H., Orozco Barrios C.E., Morales Corona J. (2024). Enhanced Neuronal Differentiation Using Plasma Synthesized Amino Polymers. Int. J. Polym. Mater. Polym. Biomater..

[28] Uribe-Juárez O., Godínez R., Morales-Corona J., Velasco M., Olayo-Valles R., Acosta-García M.C., Alvarado E.J., Miguel-Alavez L., Carrillo-González O.J., Flores-Sánchez M.G. (2021). Application of Plasma Polymerized Pyrrole Nanoparticles to Prevent or Reduce De-Differentiation of Adult Rat Ventricular Cardiomyocytes. J. Mater. Sci. Mater. Med..

[29] Zuñiga-Aguilar E., Olayo R., Ramírez-Fernández O., Morales J., Godínez R. (2014). Nerve Cells Culture from Lumbar Spinal Cord on Surfaces Modified by Plasma Pyrrole Polymerization. J. Biomater. Sci. Polym. Ed..

[30] Yue L., Lim R., Owens B.D. (2024). Latest Advances in Chondrocyte-Based Cartilage Repair. Biomedicines.

[31] Ingavle G.C., Frei A.W., Gehrke S.H., Detamore M.S. (2013). Incorporation of Aggrecan in Interpenetrating Network Hydrogels to Improve Cellular Performance for Cartilage Tissue Engineering. Tissue Eng. Part A.

[32] Islas-Arteaga N.C., Raya Rivera A., Esquiliano Rendon D.R., Morales-Corona J., Ontiveros-Nevares P.G., Flores Sánchez M.G., Mojica-Cardoso C., Olayo R. (2019). Electrospun Scaffolds with Surfaces Modified by Plasma for Regeneration of Articular Cartilage Tissue: A Pilot Study in Rabbit. Int. J. Polym. Mater. Polym. Biomater..

[33] Kilkenny C., Browne W.J., Cuthill I.C., Emerson M., Altman D.G. (2010). Improving Bioscience Research Reporting: The ARRIVE Guidelines for Reporting Animal Research. PLoS Biol..

[34] du Sert N.P., Ahluwalia A., Alam S., Avey M.T., Baker M., Browne W.J., Clark A., Cuthill I.C., Dirnagl U., Emerson M. (2020). Reporting Animal Research: Explanation and Elaboration for the ARRIVE Guidelines 2.0. PLoS Biol..

[35] Serratos I.N., Olayo R., Millán-Pacheco C., Morales-Corona J., Vicente-Escobar J.O., Soto-Estrada A.M., Córdoba-Herrera J.G., Uribe O., Gómez-Quintero T., Arroyo-Ornelas M.Á. (2019). Modeling Integrin and Plasma-Polymerized Pyrrole Interactions: Chemical Diversity Relevance for Cell Regeneration. Sci. Rep..

[36] Huang B.J., Hu J.C., Athanasiou K.A. (2016). Cell-Based Tissue Engineering Strategies Used in the Clinical Repair of Articular Cartilage. Biomaterials.

[37] Nam S., Cho W., Cho H., Lee J., Lee E., Son Y. (2014). Xiphoid Process-Derived Chondrocytes: A Novel Cell Source for Elastic Cartilage Regeneration. Stem Cells Transl. Med..

[38] Matricali G.A., Dereymaeker G., Luyten F.P. (2010). Donor Site Morbidity after Articular Cartilage Repair Procedures: A Review. Acta Orthop. Belg..

[39] Pedersen D.R., Goetz J.E., Kurriger G.L., Martin J.A. (2013). Comparative Digital Cartilage Histology for Human and Common Osteoarthritis Models. Orthop. Res. Rev..

[40] Akhtar N., Alharthi M.F., Khan M.S. (2024). Mitigating Multicollinearity in Regression: A Study on Improved Ridge Estimators. Mathematics.

[41] Aggarwal A. (2017). An Improved Parameter Estimation and Comparison for Soft Tissue Constitutive Models Containing an Exponential Function. Biomech. Model. Mechanobiol..

[42] Sophia Fox A.J., Bedi A., Rodeo S.A. (2009). The Basic Science of Articular Cartilage: Structure, Composition, and Function. Sports Health.

[43] Lotz M.K., Otsuki S., Grogan S.P., Sah R., Terkeltaub R., D’Lima D. (2010). Cartilage Cell Clusters. Arthritis Rheum..

[44] Brown E.T.T., Damen A.H.A., Thambyah A. (2020). The Mechanical Significance of the Zonally Differentiated Collagen Network of Articular Cartilage in Relation to Tissue Swelling. Clin. Biomech..

[45] Kurz B., Lange T., Voelker M., Hart M.L., Rolauffs B. (2023). Articular Cartilage-From Basic Science Structural Imaging to Non-Invasive Clinical Quantitative Molecular Functional Information for AI Classification and Prediction. Int. J. Mol. Sci..

[46] Wang W., Ye R., Xie W., Zhang Y., An S., Li Y., Zhou Y. (2022). Roles of the Calcified Cartilage Layer and Its Tissue Engineering Reconstruction in Osteoarthritis Treatment. Front. Bioeng. Biotechnol..

[47] Li J., Jiang H., Tan G., Lv Z., Liu Z., Guo H., Sun Z., Xu X., Shi D. (2025). Fibrocartilage Hyalinization: A Potential Therapeutic Strategy for Articular Fibrocartilage. J. Orthop. Transl..

[48] Ghosh S., Scott A.K., Seelbinder B., Barthold J.E., Martin B.M.S., Kaonis S., Schneider S.E., Henderson J.T., Neu C.P. (2021). Dedifferentiation Alters Chondrocyte Nuclear Mechanics during in Vitro Culture and Expansion. Biophys. J..

[49] Su W., Nie Y., Zheng S., Yao Y. (2025). Recent Research on Chondrocyte Dedifferentiation and Insights for Regenerative Medicine. Biotechnol. Bioeng..

[50] Maier F., Drissi H., Pierce D.M. (2017). Shear Deformations of Human Articular Cartilage: Certain Mechanical Anisotropies Apparent at Large but Not Small Shear Strains. J. Mech. Behav. Biomed. Mater..

[51] Yuh C., O’Bryan C.S., Angelini T.E., Wimmer M.A. (2021). Microindentation of Cartilage before and after Articular Loading in a Bioreactor: Assessment of Length-Scale Dependency Using Two Analysis Methods. Exp. Mech..

[52] Zhang H., Feng J., Zhi J., Deng Y., Zou R., Fu L., Jiang H. (2026). The Application of Tissue Engineering in Cartilage Regeneration: Technological Advances and Future Challenges. Front. Bioeng. Biotechnol..

[53] Lara-Bertrand A.L., Lizarazo-Fonseca L., Correa-Araujo L., Salguero G., Silva-Cote I. (2026). Innovative Technologies for Articular Cartilage Repair: Research, Development, and Clinical Translation—A Narrative Review. J. Funct. Biomater..

